# Age-related prevalence and imaging characteristics of the superior acetabular roof notch in children, adolescents, and young adults

**DOI:** 10.1007/s00256-023-04370-z

**Published:** 2023-06-01

**Authors:** Tobias Johannes Dietrich, Desiree Vaeth, Simon Wildermuth, Stephan Waelti, Sebastian Leschka, Nicole Graf, Tim Fischer

**Affiliations:** 1 Radiology and Nuclear Medicine, Kantonsspital St. Gallen, Rorschacherstrasse 95, 9007 St. Gallen, Switzerland; 2 Faculty of Medicine, University of Zurich, Pestalozzistrasse 3, 8091 Zurich, Switzerland; 3 Clinical Trials Unit, Kantonsspital St. Gallen, Rorschacher Strasse 95, 9007 St. Gallen, Switzerland

**Keywords:** Anatomic variant, Magnetic resonance imaging, Radiographs, Hip, Acetabulum

## Abstract

**Objective:**

To determine the age-related prevalence and imaging characteristics of the superior acetabular roof notch (SARN) on hip MRI and radiographs in a young study population.

**Materials and methods:**

Retrospective analysis of 304 MRI examinations and corresponding available radiographs of patients between the ages of 4 and 24 years. Two observers classified SARN with fluid-like findings on MRI as type-1, whereas SARN with fat-like findings on MRI were classified as type-2. Sensitivity and specificity of radiographic SARN findings were determined using MRI as the reference standard. Logistic regression models were used to assess the age-related prevalence on MRI.

**Results:**

Twelve patients (3.9%) had fluid-like SARN type-1, 27 patients (8.9%) had fat-like SARN type-2, while 265 patients (87.2%) had no SARN on MRI. The odds ratio (OR) for age (years) with respect to the presence of a fluid-like SARN type-1 on MRI was 0.79 (95% CI: 0.70–0.89), meaning that with each year, the likelihood for SARN type-1 decreased by 21% (*p* < 0.001). The OR for age with respect to the presence of a fat-like SARN type-2 on MRI was 1.14 (95% CI: 1.02–1.27) (*p* = 0.017). The diagnostic sensitivity for detecting a SARN on radiographs compared to MRI as the reference standard was between 0.75 and 0.83 and the corresponding specificity was between 0.85 and 0.89 for both observers.

**Conclusion:**

SARN is a common finding on MRI and radiographs. The present data suggest that SARN undergoes an age-related imaging characteristic from a fluid-like appearance to a fat-like appearance on MRI during adolescence.

## Introduction

Anatomic variants of the hip joint such as sublabral recess, synovial folds, stellate crease, and supraacetabular fossa can mimic labral tears, intra-articular adhesions, and chondral defects. Therefore, knowledge and identification of anatomic variants of the hip joint are important in daily clinical practice to recognize potential imaging pitfalls [1, 2]. A less commonly described anatomic variant that mimics acetabular chondral defects is the superior acetabular roof notch (SARN), which is visible on radiographs, magnetic resonance imaging (MRI), and computed tomography (CT) [1, 3–5]. The superior acetabular roof notch was described by Johnstone et al. as “a radiologic discontinuity in the medial aspect of the acetabular roof” on radiographs [3]. The authors analyzed dried skeletal specimens on radiographs and found that the anatomic explanation for the SARN was an accessory fossa in the apex of the acetabulum [3]. It was stated that neither a vascular structure passings through the SARN nor any known function could be attributed to the SARN. In addition, the ligamentum teres attaches inferomedially to the SARN and the articular cartilage does not cover the SARN. Johnstone et al. speculated that the SARN is a developmental variant of the acetabulum [3].

Recent research has shown that another anatomical variant of the acetabulum such as the supraacetabular fossa demonstrates an age-related prevalence and imaging characteristics during adolescence [6]. To our knowledge, the MR imaging characteristics of the SARN have not been reported in the literature so far.

The purpose of this study was to evaluate the imaging characteristics of the superior acetabular roof notch (SARN) on MRI and radiographs and to determine a possible age-related prevalence in a young study population.

## Materials and methods

### Patients

Institutional review board approval was obtained for this retrospective study. The local RIS and PACS databases of a tertiary general hospital and a children’s hospital serving more than 1,000,000 individuals were queried for hip MRI examinations with and without intravenous or intra-articular contrast-agent administration during an 11-year time period from 2010 to 2020. Patients without informed consent were excluded as well as patients younger than 4 years old and patients older than 25 years old. It was hypothesized that due to skeletal immaturity, a potential SARN may not be visible in the first few years of life. During the review of our data, the youngest patient with a clearly visible SARN on MRI was 4 years old, and this age was chosen as the lower inclusion age limit. The upper inclusion age limit was chosen based on the knowledge that the last growth plate of the clavicle closes in the mid-20 s and that ossification of the hip is complete between the ages of 20 and 25 years [7, 8]. Hip conditions comprising the articular cartilage and osseous structures of the hip joints were excluded, particularly, inflammatory arthritis (*n* = 1), osteoarthritis (*n* = 2), developmental dysplasia of the hip (*n* = 59), slipped capital femoral epiphysis (*n* = 2), and Perthes disease (*n* = 5). Moreover, MRI examinations with inappropriate image quality due to motion artifacts (*n* = 4) and MR images without patient’s informed consent (*n* = 5) were excluded. Out of 323 patients fulfilling these conditions, a total of 19 MRI examinations of patients with multiple MRI examinations were excluded. Finally, 304 patients were included, with a mean patient age of 17.9 ± 4.6 years and an age range of 4 to 24 years (Table 1). A total of 146 (48%) were female patients and 158 (52.0%) were male patients. MRI images of either the right or left hip were retrieved in 250 patients. MRI images of both hips were retrieved in 54 patients. Patients underwent MRI examinations between October 8, 2010, and September 30, 2020. If pelvic radiographs were available prior or after 180 days of the corresponding MRI, then the radiographs were included in the analysis of diagnostic accuracy (sensitivity and specificity). Thus, a total of 133 radiographs were included in the diagnostic accuracy analysis.

**Table 1:** Study population characteristics

Variable	Overall
Number of MRI examinations	*n* = 304
Patients’ age (mean ± standard deviation)	17.9 ± 4.6 years
Female gender	*n* = 146 (48%)
Male gender	*n* = 158 (52.0%)
MRI examinations	
Bilateral MRI	*n* = 54 (17.8%)
Unilateral MRI exclusively	*n* = 250 (82.2%)
Superior acetabular roof notch (SARN)	
No SARN	*n* = 265 (87.2%)
SARN type-1	*n* = 12 (3.9%)
SARN type-2	*n* = 27 (8.9%)
SARN on bilateral MRI	
No SARN on bilateral MRI	*n* = 48 (88.9%)
Bilateral SARN type-1 on bilateral MRI	*n* = 2 (3.7%)
Bilateral SARN type-2 on bilateral MRI	*n* = 1 (1.9%)
Unilateral SARN type-2 on bilateral MRI	*n* = 3 (5.6%)

### Imaging

MR images of all patients were obtained with a 1.5-T or 3-T MRI scanner (Magnetom Avanto fit, Magnetom Aera, Magnetom Skyra, Magnetom Skyra fit, Magnetom Vida, Siemens Healthineers, Erlangen, Germany). Various standardized MRI protocols with or without intravenous or intra-articular contrast-agent administration were used, depending on the specific clinical scenario. In general, MRI protocols included T1-weighted sequences without fat suppression as well as fluid-sensitive sequences such as STIR images, T2-weighted, or intermediate-weighted images with or without fat suppression in multiple imaging planes. For example, the 3-T MR arthrography protocol (Magnetom Skyra fit) includes the following specific sequences: transverse turbo spin-echo (TSE) T1 weighted: repetition time ms [TR]/echo time ms [TE], 742/24; field of view [FOV], 180 mm; flip angle, 120°; coronal TSE T1-weighted: TR/TE, 550/12; FOV, 160 mm; flip angle, 135°; coronal TSE intermediate weighted with fat suppression: TR/TE, 4540/33; FOV, 160 mm; flip angle, 150°; sagittal TSE intermediate weighted with fat suppression: TR/TE, 4600/33; FOV, 160 mm; flip angle, 150°; transverse oblique 3D water-excitation true fast imaging with steady-state precession: TR/TE, 11.7/5.85; FOV, 160 mm; flip angle, 30°.

Digital radiography systems (DigitalDiagnost VM, Koninklijke Philips N.V., Best, The Netherlands) with a 35 × 43 cm flat panel detector were used to obtain pelvic radiographs in anteroposterior projection with the patient in the supine position. The detector-to-tube distance was 115 cm. Grid use, tube voltage, and tube current were individually adjusted to patients age, height, and weight.

### Image evaluation

**Fig. 1:**
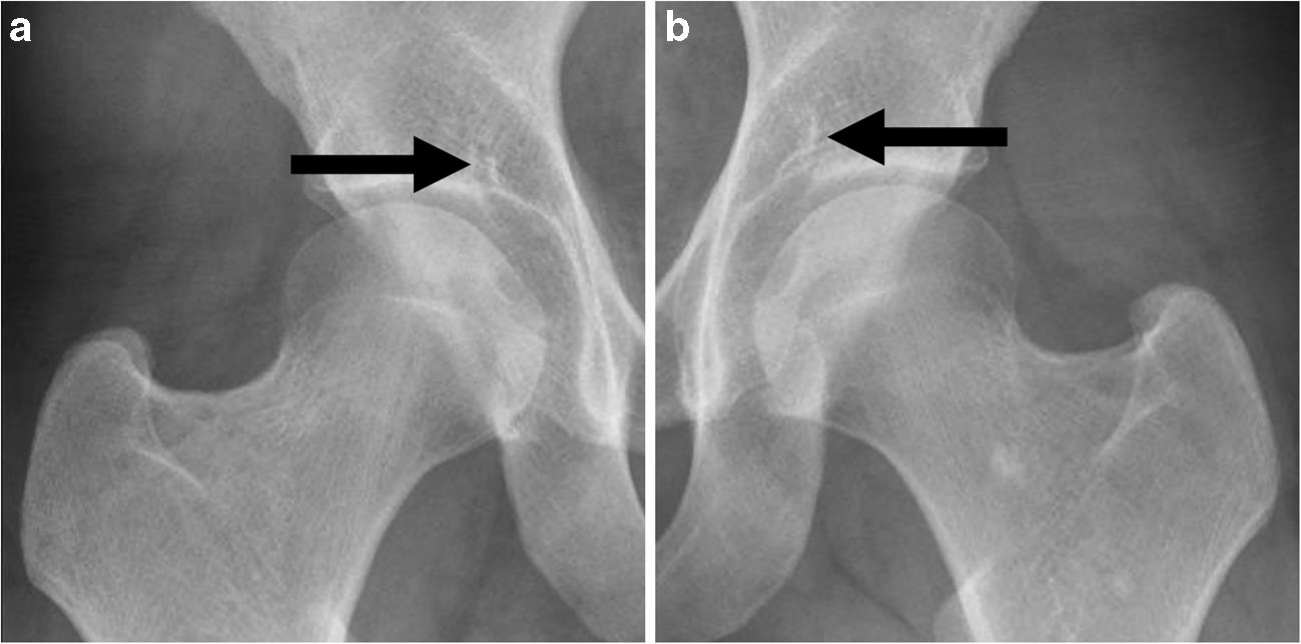
Twenty-four-year-old female. Anteroposterior radiographs show bilateral superior acetabular roof notches (SARN) (arrows) (**a** right hip; b, left hip). The SARN was originally described as “a radiologic discontinuity in the medial aspect of the acetabular roof” on radiographs [3]

**Fig. 2:**
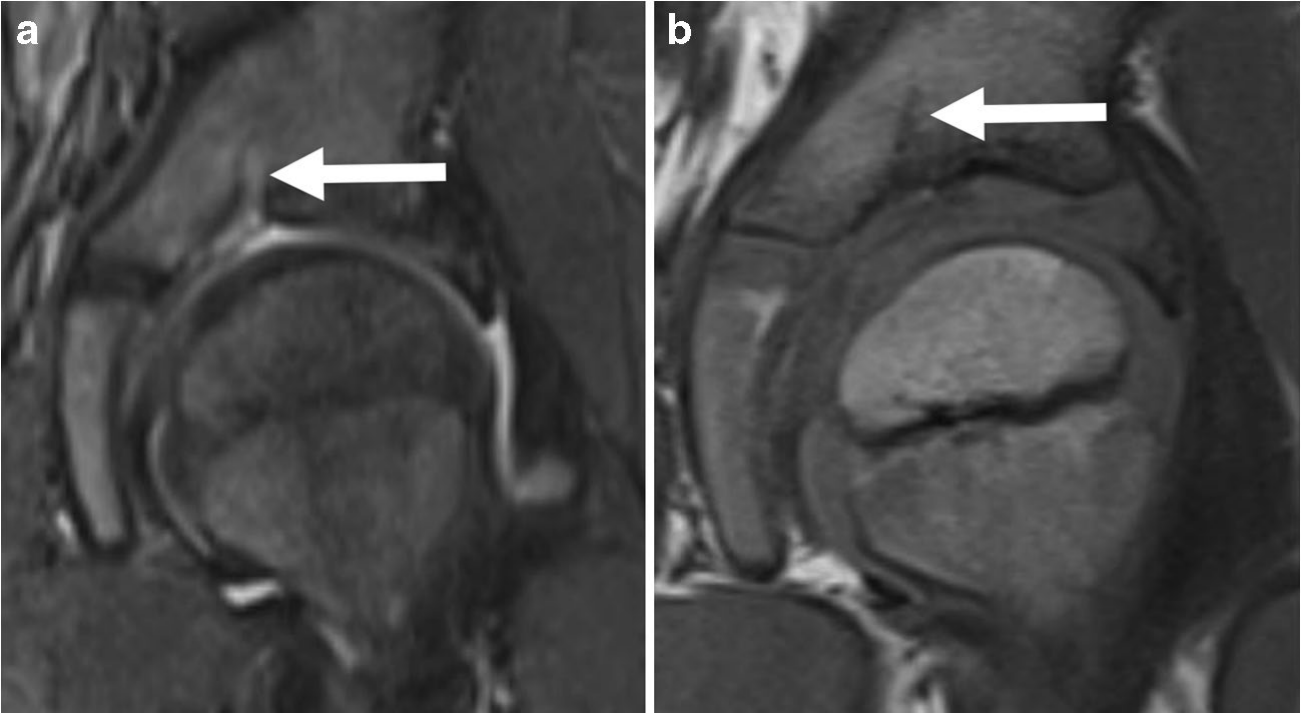
Five-year-old male. Coronal short tau inversion recovery (**a**) and coronal T1-weighted (**b**) MR images show fluid-like findings within the SARN (arrows) of the left hip, classified as a SARN type-1

**Fig. 3:**
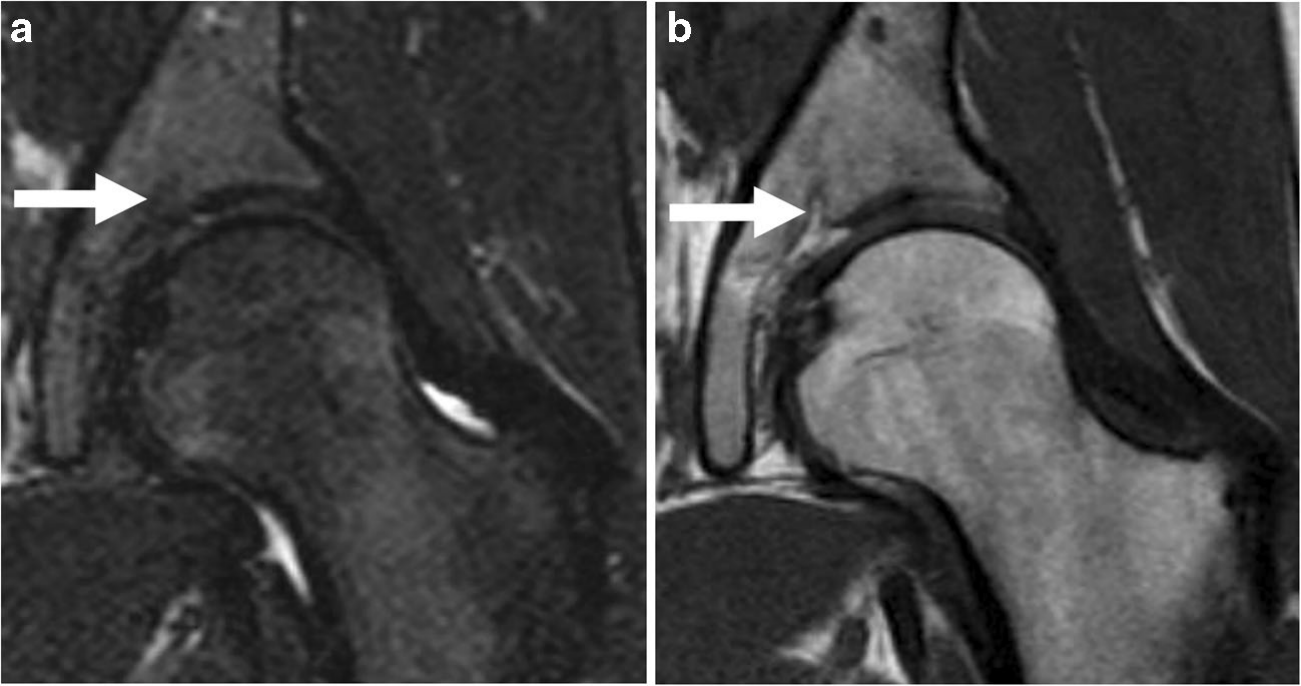
Seventeen-year-old female. Coronal short tau inversion recovery (**a**) and coronal T1-weighted (**b**) MR images show fat-like findings within the SARN (arrows) of the left hip, classified as a SARN type-2

The imaging characteristics of the SARN on radiographs have been described by Teichert and Johnstone et al. [3, 5]. As mentioned above, the MR imaging characteristics of the SARN have not been reported in the literature so far. On radiographs, the SARN has been reported as a discontinuity, a linear structure slightly converging in the superomedial direction, and a notch of the superomedial quadrant of the acetabulum [3, 5]. Imaging examples of superior acetabular roof notches on radiographs are shown in Fig. 1. According to the radiographic imaging characteristics, the present study identified the SARN on MRI as a discontinuous, linear structure slightly converging in the superomedial direction and notch of the superomedial quadrant of the acetabulum. Fluid-like findings within the SARN on MRI were classified as SARN type-1, whereas SARNs with fat-like findings within the SARN on MRI were classified as SARN type-2. Imaging examples of superior acetabular roof notches on MRI are shown in Figs. 2 and 3.

In the present study, MRI was considered the reference standard for diagnosing SARN on radiographs to determine the sensitivity and specificity for detecting SARN findings on radiographs.

### Observer

Two observers (DV, fourth year radiology resident; TF, fellowship-trained musculoskeletal radiologist with 8 years of radiology experience and 2 dedicated years in musculoskeletal radiology) independently evaluated all images. Disagreements between the two observers were resolved by a third observer (TJD, fellowship-trained musculoskeletal radiologist with 10 dedicated years of experience in musculoskeletal radiology).

### Statistical analysis

Cohen’s kappa and 95% bootstrapped percentile confidence intervals (CI) with 1000 replicates were analyzed as measures of reliability. Logistic regression models were run to assess the influence of age on the presence of SARN type-1 and SARN type-2. Influential values were checked by plotting Cook’s distance. Assumptions of linearity, normality, and homoskedasticity were checked with residual plots. Sensitivity and specificity with corresponding exact 95% confidence intervals for binomial probabilities were calculated for MRI.

A statistician (NG) performed all statistical analyses using the R programming language (R Core Team, 2020, version 4.0.2) [9]. The packages “tableone,” “irr,” “boot,” and “Hmisc” were used to compute descriptive statistics, Cohen’s kappa, confidence intervals for Cohen’s kappa, as well as for sensitivity and specificity [10–13].

## Results

**Fig. 4:**
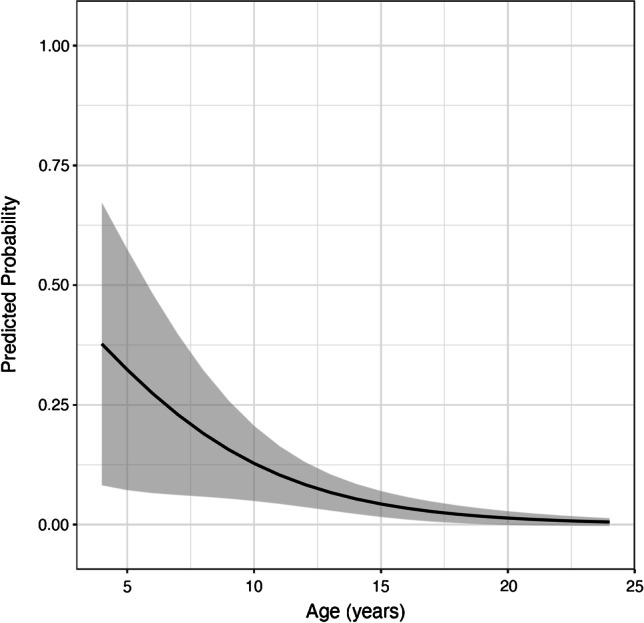
Superior acetabular notch type-1: predicted probability with 95% confidence bands of superior acetabular notch type-1. The OR was 0.79, meaning that with each year the likelihood of superior acetabular notch type-1 decreased by 21% (*p* < 0.001)

**Fig. 5:**
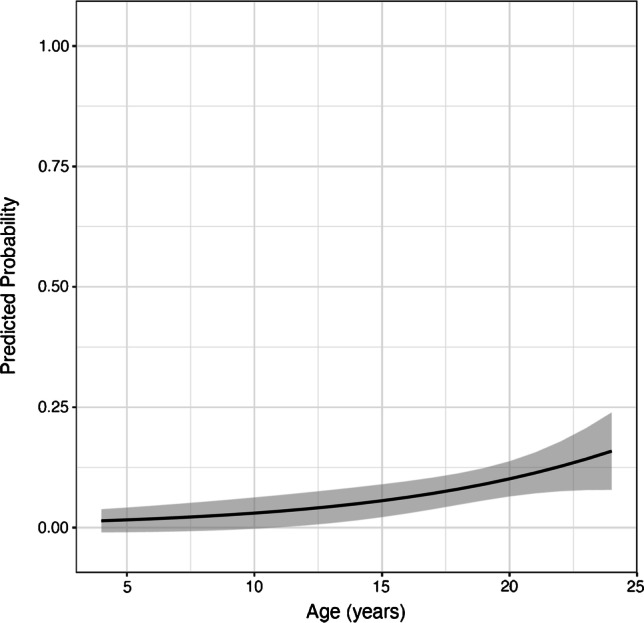
Superior acetabular notch type-2: predicted probability with 95% confidence bands of superior acetabular notch type-2. The OR was 1.14, meaning that with each year the likelihood of superior acetabular notch type-2 increased by 14% (*p* = 0.017)

Twelve patients (3.9%) had a fluid-like SARN type-1, 27 patients (8.9%) had a fat-like SARN type-2, and 265 patients (87.2%) had no SARN on MRI. The interobserver reliability analysis for the detection of SARN on MRI showed a kappa value of 0.61 (95% CI: 0.47–0.74), and for the detection of SARN on radiographs, a kappa value of 0.73 (95% CI: 0.58–0.86). The inclusion of female and male gender as a predictor in the models for SARN type-1 and type-2 did not improve the fit of the models; thus, the models were presented without gender-related analyses. The odds ratio (OR) for age (years) with respect to the presence of a fluid-like SARN type-1 on MRI was 0.79 (95% CI: 0.70–0.89), meaning that with each year, the likelihood of SARN type-1 decreased by 21% (*p* < 0.001) (Table 2, Fig. 4). The OR for age with respect to the presence of a fat-like SARN type-2 on MRI was 1.14 (95% CI: 1.02–1.27), meaning that with each year, the likelihood of SARN type-2 increased by 14% (*p* = 0.017) (Table 3, Fig. 5).

**Table 2:** SARN type-1: logistic regression model with age as a predictor of superior acetabular notch type-1. The OR was 0.79, meaning that with each year the likelihood for superior acetabular notch type-1 decreased by 21% (*p* < 0.001)

	OR	Lower 95% confidence limit	Upper 95% confidence limit	*p*-value
(Intercept)	1.56	0.28	8.58	0.609
Age	0.79	0.70	0.89	< 0.001

**Table 3:** SARN type-2: logistic regression model with age as a predictor of superior acetabular notch type-2. The OR was 1.14, meaning that with each year the likelihood for superior acetabular notch type-2 increased by 14% (*p* = 0.017)

	OR	Lower 95% confidence limit	Upper 95% confidence limit	*p*-value
(Intercept)	0.009	0.001	0.072	< 0.001
Age	1.14	1.02	1.27	0.017

The diagnostic sensitivity for detecting a SARN on radiographs compared to the reference standard, MRI, was 0.75 (95% CI: 0.43–0.95) and 0.83 (95% CI: 0.52–0.98), and the corresponding specificity value was 0.85 (95% CI: 0.78–0.91) and 0.89 (95% CI: 0.82–0.94) for both observers.

## Discussion

This study demonstrated that the superior acetabular roof notch is a common variant on MRI and radiographs. Twelve patients (3.9%) had a fluid-like SARN type-1, 27 patients (8.9%) had a fat-like SARN type-2, and 265 patients (87.2%) had no SARN on MRI. The present article found a prevalence of approximately 13% for the superior acetabular roof notch on MRI. The present data suggest that SARNs undergo an age-related imaging characteristic from a fluid-like appearance to a fat-like appearance on MRI during adolescence.

The imaging findings of the SARN on radiographs and CT images have been briefly reported previously in both review and original articles [1, 3, 5, 6]. To our knowledge, neither the MR imaging findings of the SARN nor the prevalence of the SARN on radiographs, CT, or MR imaging has been systematically described so far in the literature.

More than 60 years ago, Teichert reported the SARN as an unusual linear structure of the superomedial quadrant of the acetabulum slightly converging in the superomedial direction [5]. Approximately 40 years ago, Johnstone et al. redefined and specified Teichert’s work. These authors compared dried skeletal anatomic specimens with their radiographic appearance and reported an apparent radiographic discontinuity of the superomedial portion as an anatomic fossa and renamed the SARN as the superior acetabular roof notch and “superior acetabular notch” [3]. It has been noted that the SARN has no known function, specifically the SARN is not a vascular structure, is commonly observed on radiographs in normal subjects, and can be considered an anatomic variant [3, 5].

Pseudodefects of the acetabular cartilage are variants, can mimic chondral defects, and do not require any treatment. These pseudodefects of the acetabular cartilage such as the supraacetabular fossa, stellate crease, and superior acetabular roof notch are frequently presented in review articles and uncommonly analyzed in original articles [1, 3–6]. The supraacetabular fossa was described as an accessory osseous fossa in the acetabular roof filled with cartilage or synovial fluid and clearly distinguishable by the acetabular fossa. The reported prevalence of the supraacetabular fossa was between 11 and 36% [1, 6, 14]. Interestingly, an age-related phenomenon was observed for the supraacetabular fossa: The supraacetabular fossa was most frequently observed in 14-year-old adolescents. In young patients the prevalence of the supraacetabular fossa decreased beyond 14 years. The prevalence of the SAF initially increased until 14 years of age; in older patients the prevalence and size of the supraacetabular fossa decreased again. Moreover, it was concluded that during skeletal maturation, the supraacetabular fossa develop from a fluid-filled fossa to a cartilage filled to no fossa [6].

The stellate crease is a starlike hyaline cartilage-deficient transient zone between the acetabular notch and acetabular roof [1, 4]. On arthroscopy the stellate crease presents with linear indentations of the hyaline cartilage above the acetabular roof and may be misinterpreted as early cartilage degeneration. The appearance and prevalence of the stellate crease on radiographs, CT, CT arthrography, MRI, or MR arthrography have not been described in original articles yet [4]. Particularly, a transition from a fluid-like finding of the stellate crease or supraacetabular fossa to a fat-like finding on MRI during adolescence has not been presented in the literature. We did not find a similar phenomenon of anatomic variants with a development from a fluid-like to a fat-like finding on MR images.

Another fluid-like pseudodefect of the hyaline cartilage of a concave articular surface in the body is the glenoid bare spot in the shoulder. Like the supraacetabular fossa, the highest prevalence of the glenoid bare spot was observed at 14–15 years of age in a study population consisting of children, adolescents, and young adults. Based on the bell-shaped age-related prevalence curve of the of glenoid bare spot, the authors suggested a developmental etiology for the glenoid bare spot [15].

A limitation of this work is the retrospective study design. Neither gross anatomic specimens nor open surgery or arthroscopy were used as reference standards. The present study was conducted at hospitals in Eastern Switzerland. Thus, we speculate that the present study cohort represent the typical population of Eastern Switzerland, Central Europe. Approximately 25% of the Eastern Swiss population are non-Swiss citizens, most of them are from Central Europe, Southern Europe, and Southeastern Europe. Smaller groups of non-Swiss citizens in Eastern Switzerland are from Western Asia, Central Asia, Africa, and Western Europe [16, 17]. Evaluation of differences between ethnicities of the present study cohort is a very challenging topic because patients were not assigned to ethnic groups in the involved hospitals, neither in the hospital information system nor in the radiological information system. Thus, this retrospective study cannot compare the prevalence and imaging characteristics of the superior acetabular roof notch to the various ethnicities.

In conclusion, superior acetabular roof notch is a common finding on MRI and radiographs. The present data suggest that the superior acetabular roof notch undergoes an age-related imaging characteristic from a fluid-like appearance to a fat-like appearance on MRI during adolescence.

## Data Availability

The dataset analyzed during this study are available from the corresponding author on reasonable request.

## Abbreviations

CI: Confidence interval
CT: Computed tomography
FOV: Field of view
MRI: Magnetic resonance imaging
OR: Odds ratio
SARN: Superior acetabular roof notch
TE: Echo time
TR: Repetition time
TSE: Turbo spin echo
